# Children Facing the Unknown: An Italian Study Using the Intolerance of Uncertainty Scale– Parent (IUS-P)

**DOI:** 10.1007/s10802-025-01355-5

**Published:** 2025-08-05

**Authors:** Sara Iannattone, Lisa Toffoli, Alessandra Farina, Giovanni Mento, Gioia Bottesi

**Affiliations:** 1 Department of General Psychology, University of Padova, via Venezia 8, Padova, 35131 Italy; 2 Scientific Institute, IRCCS E. Medea, Conegliano, Treviso, Italy

**Keywords:** Assessment, Childhood, Executive function, Intolerance of uncertainty, Psychometrics, Psychopathological features

## Abstract

Research on Intolerance of Uncertainty (IU) has predominantly focused on adulthood, with limited attention to children. However, examining IU during childhood is clinically important, as it can shed light on the development of early-emerging psychopathologies. Valid and reliable tools assessing IU in children are therefore essential. This study aimed to investigate the factor structure and psychometric properties of the Intolerance of Uncertainty Scale-Parent (IUS-P), a questionnaire for parents to evaluate IU in their children. 796 Italian parents (88.4% mothers) of children aged 4–10 years (51.3% boys) were involved. Other-report tools assessing IU, psychopathological features, and executive functioning impairments (i.e., cognitive inflexibility, impulsivity, difficulties in planning/organization, and emotion dysregulation) were administered. To investigate the IUS-P factor structure, we compared a one-factor model, a two-factor model, and a bifactor model. Measurement invariance across sex and age groups (i.e., 4–6 vs. 7–8 vs. 9–10 years), reliability, and construct validity were evaluated. The bifactor model outperformed competing factor models. Bifactor model indices suggested using a total score instead of separate subscale scores. Measurement invariance by sex and age groups was fully supported. Additionally, the IUS-P total score exhibited excellent one-month test-retest reliability. The total score also showed positive associations with psychopathological features and executive functioning impairments. These findings highlight the IUS-P as a reliable tool to evaluate IU in Italian children. The relations of IU with psychopathological dimensions and difficulties in executive functioning underscore the transdiagnostic nature of IU during childhood. The practical implications of these results are discussed.

**Supplementary Information:**

The online version contains supplementary material available at 10.1007/s10802-025-01355-5.

Intolerance of Uncertainty (IU) is a dispositional trait reflecting the tendency to be bothered by the (still) unknown elements of a particular situation, regardless of whether the potential outcome is negative (Freeston et al., 2020). Individuals with high levels of IU exhibit significant difficulty in tolerating and modulating the negative emotions triggered by uncertainty, a phenomenon known as uncertainty distress (Freeston et al., 2020). IU was initially conceptualized as a key factor in the onset and maintenance of worry, the core feature of Generalized Anxiety Disorder (GAD) (Dugas et al., 1998). While IU remains central to the understanding of GAD, current literature increasingly positions it as a transdiagnostic factor implicated in various psychopathologies (e.g., Bottesi et al., 2018, 2021; Gentes & Ruscio, 2011; Iannattone et al., 2022; Oglesby et al., 2016). Notably, most evidence supporting the transdiagnostic nature of IU focuses on adulthood, despite the fact that the fundamental cognitive skills required to perceive and respond to uncertainty appear to develop during childhood (Goupil et al., 2016). This notwithstanding, much of the research examining the relation between IU and psychopathological constructs in developmental age has centered on adolescents (aged 11–17 years) or young individuals spanning wide age ranges, from 4 to 18 years old (Osmanağaoğlu et al., 2018). In contrast, relatively few studies have specifically focused on school/preschool-aged children (4–11 years). Specifically, existing research in clinical child samples has linked IU to the symptomatology of anxiety (e.g., Glod et al., 2019), Autism Spectrum Disorder (ASD) (e.g., Cardon et al., 2023; Ozsivadjian et al., 2021), and Attention Deficit and Hyperactivity Disorder (ADHD) (Gramszlo et al., 2018). Interestingly, these conditions are often characterized by executive functioning difficulties, suggesting a potential link between aspects of executive functioning - such as emotion regulation - and heightened IU (e.g., Gramszlo et al., 2018). However, studies specifically targeting non-clinical school/preschool-aged children remain scarce, although exploring IU in this population could provide valuable insights into the developmental trajectories of psychopathologies that emerge during childhood. To achieve this goal, valid and reliable assessment tools for IU in this developmental stage are needed.

## Assessment of IU During Developmental age

Most research on IU measurement has focused on adults and/or young adults (university students) (Bottesi et al., 2020). Specifically, much attention has been given to the Intolerance of Uncertainty Scale-12 (IUS-12; Carleton et al., 2007), a questionnaire that represents the shortened version of the original 27-item Intolerance of Uncertainty Scale (IUS-27; Freeston et al., 1994). The IUS-12 is the most widely used self-report measure for assessing IU in adulthood (Bottesi et al., 2019; McEvoy et al., 2019). Initial research on the factorial structure of the IUS-12 has suggested that it consists of two distinct dimensions: Prospective IU, which refers to the predisposition to actively seek information to reduce uncertainty, and Inhibitory IU, which expresses avoidance-oriented reactions to uncertainty (Birrell et al., 2011; Carleton et al., 2007; McEvoy & Mahoney, 2011). However, recent literature favors a general factor over the two canonical factors and supports using the total score instead of two highly intercorrelated subscale scores (Bottesi et al., 2019; Huntley et al., 2020; Wilson et al., 2020). Boelen et al. (2010) have been the first to analyze the factorial structure of the IUS-12 in adolescents (14–18 years old). The study revealed a two-factor structure (Prospective IU and Inhibitory IU) similar to that found in adults (Carleton et al., 2007). However, this research has some limitations: it did not include participants under 14 years of age and used the IUS-12 instead of a measurement tool specifically created for younger individuals.

### The Intolerance of Uncertainty Scale for Children (IUSC)

The first attempt to develop a scale to investigate IU in children and adolescents is the Intolerance of Uncertainty Scale for Children (IUSC; Comer et al., 2009). The IUSC is a derivative version of the IUS-27 (Freeston et al., 1994) adapted for individuals aged 7 to 17 years old (Comer et al., 2009). It comprises two parallel modules, each consisting of 27 items: a self-report module and an other-report module, the latter intended to be completed by parents to evaluate their children’s tendency to react negatively to uncertain situations (Comer et al., 2009). In the self-report version, item formulations of the original IUS-27 were modified to enhance children’s understanding, with three specific objectives: (a) to reduce metacognitive content and references requiring a sophisticated understanding of mental processes, (b) to eliminate figurative language, complex phrasing, and idioms that children may not easily interpret literally, and (c) to decrease the number of polysyllabic words (i.e., 3 syllables) (Comer et al., 2009). In the parent-report version of the IUSC, instead, items were adapted from the child-report version to prompt parents to rate their child’s tendency to react negatively to uncertain situations and events (Comer et al., 2009).

The psychometric properties of the IUSC have been initially evaluated in a study by Comer et al. (2009) which included 197 young people aged between 7 and 17 years old, both with and without anxiety disorders. The total score exhibited excellent internal consistency in both samples. However, due to the number of items in the IUSC, the sample sizes did not allow for factor analysis (Comer et al., 2009). More recently, Cornacchio et al. (2018) proposed a shortened 12-item version of the IUSC (IUSC-12), whose factorial structure was tested in a sample of young people aged 9 to 18 years old, again including a mixed sample of individuals with and without anxiety disorders. The results indicated the presence of multiple acceptable factorial structures, including both a two-factor model (Prospective IU and Inhibitory IU) and a bifactor one (Hale et al., 2016). While the latter demonstrated a better fit to the data, concurrent validity analyses showed that the Prospective and Inhibitory IU factors were capable of predicting anxiety and depression scores, rendering them particularly useful in clinical settings (Cornacchio et al., 2018). Lastly, Osmanağaoğlu et al. (2021) tested both versions of the IUSC (i.e., 27 and 12 items) in a non-clinical sample of children and preadolescents (age range: 7–11 years old). Specifically, both modules (self-report for children and other-report for parents) of the two versions of the IUSC were utilized. The 27-item IUSC (both self-report and other-report) demonstrated inadequate fit indices. Regarding the self-report IUSC-12, instead, results revealed that a one-factor model with 11 items fitted the data better than a two-factor model; however, both models were equivalent in terms of fit and parsimony when item #10 was retained (Osmanağaoğlu et al., 2021). Finally, concerning the other-report IUSC-12, only the one-factor structure was supported with all 12 items retained. Of note, a weakness of the IUSC is that various adaptations of the same questionnaire for children and adults may result in different items and structures. This can often complicate the interpretation and comparison of scores obtained from different populations (Bottesi, 2023).

### The Intolerance of Uncertainty Scale-Parent (IUS-P)

Almost simultaneously with the IUSC, Walker et al. (2010) proposed the Intolerance of Uncertainty Scale-Revised (IUS-R), which represents a refined version of the IUS-12 (Carleton et al., 2007). The IUS-R was designed with simplified language to be easily understood by an average 11-year-old student (Walker et al., 2010). It serves as a self-report tool for assessing IU across the lifespan and facilitates the integration of results from different studies (Boulter et al., 2014). The original version of the IUS-R has been used with both non-clinical children and adolescents, as well as those diagnosed with ASD or Asperger’s syndrome (e.g., Boulter et al., 2014; Joyce et al., 2017). Regarding the factorial structure, preliminary results from a study by Bottesi and Freeston (2012) involving eight British and Spanish non-clinical groups (ages 6–8 years old, 9–11 years old, 12–14 years old, and university students) indicated that the two-factor model adequately fitted the data; however, a bifactor model was not tested. In the Italian context, Bottesi et al. (2023) investigated the factorial structure and psychometric properties of the IUS-R in a non-clinical group of adolescents (age: 11–17 years). Their findings supported the bifactor structure of the questionnaire, particularly highlighting the presence of a general IU factor and two specific factors corresponding to Prospective IU and Inhibitory IU.

In response to the need for a measure that would allow one to obtain other-reported information about IU in children, while also enabling easy comparison with the results obtained from older age groups, the Intolerance of Uncertainty Scale-Parent (IUS-P; Rodgers et al., 2012) was developed. Specifically, it is a readaptation of the IUS-R, containing the same 12 items but intended to be completed by parents thinking about their children. The IUS-P has been utilized to investigate IU in various samples of children and adolescents aged 4 to 18 years old with ASD (e.g., Boulter et al., 2014; Rodgers et al., 2023; Wigham et al., 2015). In all cases, a significant relation between IU, anxiety, and ASD has been confirmed. Nevertheless, despite its use in several studies, no one has yet tested the factorial structure and psychometric properties of the IUS-P.

### The Current Study

Building on the aforementioned state of the art, the present study mainly aimed to provide data on the factorial structure and psychometric properties of the IUS-P in a large sample of Italian parents of neurotypical children aged 4 to 10 years. Notably, previous studies on the assessment of IU in childhood have either focused on children aged 7 years and older or included younger children (from age 4), but only within clinical samples. By contrast, the present study extends the existing literature by assessing IU in early childhood within a non-clinical population, thereby offering a broader and developmentally nuanced perspective on this construct and its measurement.

From a practical perspective, a valid and reliable measure of IU in children is crucial, since this construct has been increasingly recognized as a key factor associated with psychological issues in developmental age (e.g., Bottesi, 2023; Iannattone et al., 2023; Osmanağaoğlu et al., 2018). Nevertheless, given that children aged 4–10 years are still developing cognitive and emotional capacities, they may struggle to fully comprehend or accurately report on their internal experiences, especially when it comes to complex psychological constructs like IU. In contrast, parents are often the most reliable observers of their child’s emotional and behavioral responses in everyday situations, placing them in a unique position to provide accurate reports on their child’s tendencies to experience and react to uncertainty. As a result, parent-rated measures, such as the IUS-P, offer a practical and valuable means of capturing IU in children. In addition, and importantly, parents are typically involved in the assessment phase of psychological interventions with children, making parent-report tools especially useful in clinical practice. To ensure accurate assessment of children’s IU, however, establishing the psychometric properties of the IUS-P is a critical first step; indeed, without this validation, the tool’s suitability for measuring IU in children could be questioned, limiting its value in both research and clinical settings.

Given that the IUS-P is derived from the IUS-R, our hypotheses on the IUS-P factor structure were guided by existing literature on the IUS-R in non-clinical samples. First, we hypothesized that the bifactor structure would be confirmed, supporting the use of the IUS-P total score for assessment purposes (Bottesi et al., 2019, 2023). To be specific, in bifactor modeling, each item is specified to load onto both a general factor - reflecting the common variance shared across all items - and a specific factor corresponding to its subscale. In this framework, specific factors are uncorrelated with one another, and the general factor is orthogonal to all specific factors (Hale et al., 2016). Subsequently, we investigated the measurement invariance of the IUS-P factorial structure across sex and age groups, specifically differentiating between preschoolers (4–6 years), young school-aged (7–8 years), and middle school-aged (9–10 years) children. Furthermore, the psychometric properties of the IUS-P were analyzed in terms of internal consistency, convergent validity, and one-month test-retest reliability. With regard to convergent validity, the IUS-P was expected to demonstrate moderate-to-strong positive correlations with measures of internalizing features, in line with previous studies on child (Osmanağaoğlu et al., 2018; Ryan et al., 2025) and adolescent (Bottesi et al., 2023) samples. Moreover, we hypothesized to find weak-to-moderate positive correlations between the IUS-P and measures of externalizing features. This last hypothesis was mainly exploratory, as - to our knowledge - no study to date has specifically examined the associations between IU and distinct externalizing domains in children. This lack of prior literature prevented us from formulating more fine-grained hypotheses on this matter. Instead, we relied on broader expectations drawn from the Italian validation study of the IUS-R among adolescents, which used the same externalizing dimensions (albeit via adolescent self-report) and found weak-to-moderate associations (Bottesi et al., 2023). Crucially, as a secondary and exploratory aim, we also examined the associations between the IUS-P and measures of executive functioning, addressing an underexplored area in developmental research. Notably, examining this association may provide insights into whether IU is, at least in part, linked to executive functioning difficulties. To be specific, we focused on key domains of executive functioning previously examined in relation to IU in adults or adolescents, though not typically framed within the context of executive functioning itself. These domains included: cognitive flexibility (e.g., Gabrys et al., 2018), the ability to manage current and future-oriented task demands (e.g., Hromova, 2022), emotion regulation (e.g., Lauriola et al., 2023), and inhibitory control/impulsivity (e.g., Bottesi et al., 2018). Although we anticipated positive associations between IU and difficulties across the above executive functions, we refrained from making specific predictions regarding the strength of these associations due to the lack of previous research directly examining IU and executive functioning in children. Of note, understanding the link between IU and executive functioning in children is valuable, as it may offer new insights into the developmental mechanisms underlying IU and its bidirectional impact on executive functioning. In addition, similarly to IU, executive dysfunction is commonly associated with psychopathology in young people, particularly regarding the severity and chronicity of psychological problems (Halse et al., 2022). Moreover, both IU and executive dysfunction generally characterize neurodevelopmental conditions like ADHD and ASD (e.g., Gramszlo et al., 2018; Jenkinson et al., 2020). Consequently, investigating the interplay between IU and executive functioning impairments may help refine interventions targeting both dimensions in a transdiagnostic perspective. This, in turn, may enhance the effectiveness of such interventions in preventing the onset of severe psychopathological conditions in children, especially in those with neurodevelopmental conditions who are at elevated risk for psychopathology (Du Rietz et al., 2021).

## Methods

### Participants and Procedure

The present study was approved by the Ethics Committee for Psychological Research of the University of Padova (N. 4739) and conducted in accordance with the Declaration of Helsinki. Before data collection, the IUS-P was translated following standard guidelines recommended in psychological research (*see* Brislin, 1986 for further details).

Data were collected from September 2022 to January 2024 in different Italian regions. The original sample consisted of 945 parents. However, 43 participants were excluded because the age of their children was out of the range considered in the present study (i.e., 4–10 years), while 54 because they reported that their children had received a diagnosis of developmental issues (e.g., ASD, hyperactivity, language impairment, learning problems, disabilities). Finally, 52 participants were excluded based on the Behavior Rating Inventory of Executive Function (BRIEF; Marano et al., 2014, 2016) scale validity criteria. The final sample consisted of 796 parents, of whom 88.4% were mothers (age range: 26–55 years, *M* = 41.6 ± 4.98) and 11.6% were fathers (age range: 31–58 years, *M* = 44.6 ± 5.65). Other socio-demographic characteristics of parents are summarized in Table S1 of the Supplementary Information (*see* Online Resource). Their children (*N* = 796; 51.3% boys) were aged 4 to 10 years (*M* = 7.53 ± 1.74). Of these, 4.9% were in their second year of kindergarten, 9.8% in their third year of kindergarten, and the rest attended elementary school: 17.8% were in first grade, 17.3% in second grade, 18.7% in third grade, 15.5% in fourth grade, and 16.0% in fifth grade. The mean raw and T-scores on all the administered questionnaires, as well as the mean IUS-P scores stratified by age range and sex are available in the Supplementary Information of the Online Resource (Tables S2 and S3, respectively).

An ad hoc online survey, consisting of a socio-demographic form and a battery of parent-report instruments (*see* list below), was administered to the sample and took approximately 45/50 minutes to be completed. Recruitment of parents occurred through controlled channels – namely, social media and lists of individuals who had previously consented to be recontacted for further research – which limited the likelihood of non-human responses. Moreover, basic attention-check procedures were embedded in the survey, including reverse-coded items, logically linked questions, and instructional manipulation checks (e.g., items asking participants to select a specific response to confirm attentiveness). Data were also screened for response patterns indicative of low engagement (e.g., uniform or random responding), and no anomalies were detected. Importantly, only one parent per child was asked to complete the survey. Parents were recontacted to complete the IUS-P one month later, with 320 participants from the initial group agreeing to this follow-up. Confidentiality of data treatment was guaranteed by using an identification code created by each participant at the beginning of the survey.

### Measures

The online survey included the following questionnaires:

- The *Intolerance of Uncertainty Scale – Parent* (IUS-P; Rodgers et al., 2012) is a 12-item questionnaire that provides parent-reported IU in children. Respondents rate the extent to which each statement is characteristic of their child on a five-point Likert scale (*1 = not at all agree*,* 5 = completely agree*). The IUS-P has been found to have excellent internal consistency in samples of neurotypical children’s parents (Cronbach’s α = 0.91, Boulter et al., 2014; Cronbach’s α = 0.88, Neil et al., 2016).
- The *Child Behavior Checklist 6–18* (CBCL 6–18; Italian version by Frigerio et al., 2004) is the parent version of the Youth Self Report 11–18 (YSR 11–18) used to assess emotional and behavioral problems in youth. It comprises 112 items rated on a three-point Likert scale (*0 = not true; 1 = sometimes true; 2 = very true*). Problem behaviors are identified through eight syndrome scales: anxiety/depression, withdrawal/depression, somatization, social problems, thought-related problems, attention problems, aggressive behavior, and rule-breaking behavior. In the Italian version of the tool, the α values were moderate to good (α > 0.65) for half of these scales; however, some scales (i.e., somatization, social problems, thought-related problems and rule-breaking behavior) demonstrated lower reliability (α < 0.65) (Frigerio et al., 2004). For the purpose of the present study, we used all syndrome scales except for the thought-related problems and rule-breaking behavior scales, which were excluded due to their insufficient internal consistency in our sample (α = 0.55 and 0.43, respectively). Cronbach’s α coefficients for the other syndrome scales were 0.79 for anxiety/depression, 0.67 for withdrawal/depression, 0.65 for somatization and social problems, 0.76 for attention problems, and 0.83 for aggressive behavior.
- The *Behavior Rating Inventory of Executive Function— Preschool* (BRIEF-P; Italian version by Marano et al., 2014) and *Behavior Rating Inventory of Executive Function – Second Edition* (BRIEF-2; Italian version by Marano et al., 2016) are designed for parents to assess distinct components of executive functioning in pre-school and school-aged children (2–5.11 years of age for the BRIEF-P and 5–18 years of age for the BRIEF-2). They consist of 63 items rated on a three-point Likert scale (i.e., *1 = never*,* 3 = often*), with higher scores indicating more difficulties in executive functioning. In this study, we decided to use only the scales included in both versions of the questionnaire: Inhibit (assessing inhibitory control and impulsivity, or the ability to resist impulses and stop behavior at the appropriate time), Shift (measuring cognitive flexibility, or the ability to adapt quicky and effectively to new situations, demands, or problem aspects), Emotional control (evaluating the impact of executive functioning problems on emotional expression and regulation), and Plan/Organize (assessing the ability to manage current and future-oriented task demands). Internal consistency for the BRIEF-P scales ranged from α = 0.76 (Plan/Organize) to α = 0.87 (Inhibit) (Marano et al., 2014). Instead, as regards the BRIEF-2, internal consistency ranged from α = 0.70 (Shift) to α = 0.88 (Emotional control) (Marano et al., 2016). In the present sample, Cronbach’s α coefficients for the BRIEF-P scales were 0.70 for Plan/Organize, 0.78 for Emotional control, 0.80 for Shift, and.84 for Inhibit. Regarding the BRIEF-2, instead, Cronbach’s α coefficients were 0.78 for Shift and Inhibit, 0.82 for Plan/Organize, and 0.85 for Emotional control. Importantly, we intentionally chose this parent-report questionnaire, as it offers an efficient way to capture parents’ perceptions of their child’s executive function based on everyday behavior (O’Meagher et al., 2019). Indeed, by gathering parent-reported observations of the child’s behaviors and symptoms in daily life, the BRIEF enables the assessment of how specific executive function components manifest in real-life contexts, thereby providing an ecologically valid measure of executive (dys)function.

### Data Analysis

A Confirmatory Factor Analysis (CFA) approach was used to evaluate the factorial structure of the IUS-P by testing three models: a one-factor model, a two-factor model with correlated factors (i.e., Prospective IU and Inhibitory IU), and a bifactor model with three orthogonal factors (i.e., Prospective IU, Inhibitory IU, and General IU). In the bifactor model, the specific factors (Prospective and Inhibitory IU) separate random error variance from the variance common to specific groups of items. The general IU factor instead captures the common variance across all items, representing the core trait underpinning IU beliefs above and beyond the specific dimensions of Prospective and Inhibitory IU. CFAs were performed using robust weighted least squares (WLSMV) estimation, appropriate for the ordinal nature of the response scale (i.e., a five-point Likert scale) (DiStefano & Morgan, 2014). Model fit was assessed using the scaled χ² statistic and other commonly recommended indices, including the Comparative Fit Index (CFI), Tucker-Lewis Index (TLI), Root Mean Square Error of Approximation (RMSEA), and Standardized Root Mean Square Residual (SRMR). CFI and TLI values greater than 0.95 indicate a good fit, while values exceeding 0.90 are considered acceptable. An RMSEA value of 0.06 or lower is considered a good fit, with values below 0.08 being acceptable. An SRMR value below 0.08 supports a good fit between the model and the data (Schermelleh-Engel et al., 2003). To determine the baseline model for subsequent measurement invariance analyses, we compared the fit of the one-factor, two-factor, and bifactor models.

In relation to the bifactor model, its appropriateness for understanding the properties of a psychometric scale can be evaluated on the basis of specific indices: the Percentage of Uncontaminated Correlations (PUC) and the Explained Common Variance (ECV) (Rodriguez et al., 2016). The PUC quantifies the proportion of item correlation coefficients that are influenced only by the variance of the general factor, while the ECV represents the proportion of variance in the items that is explained by the general factor in relation to the total shared variance. When both the PUC and ECV exceed 0.70, the scale can be considered unidimensional, and the factor loadings for the one-factor model and the general factor in a bifactor model are expected to be similar (Rodriguez et al., 2016). Additionally, the reliability of the scale can be assessed using ω and ωh coefficients. The ω coefficient estimates the overall proportion of reliable variance in the IUS-P total score accounted for by both the general and group factors, while ωh specifically reflects the proportion of reliable variance in the total score attributable to the general factor. Minimal differences between these reliability coefficients support the use of the IUS-P total score as a valid measure of IU in children.

Next, we tested measurement invariance of the best-fitting model for the IUS-P across sex and age groups (i.e., 4–6 vs. 7–8 vs. 9–10 years). Given the categorical nature of the IUS-P items, the invariance tests followed the procedure suggested by Svetina et al. (2020). First, configural invariance was tested to verify whether the same items measured the same latent constructs across groups. Then, threshold invariance was assessed to examine whether item thresholds were consistent across groups. Finally, metric invariance was evaluated to ensure that both the factor loadings and item thresholds were equivalent across groups. Threshold invariance is achieved when the model with equality constraints on item thresholds across groups fits the data as well as the configural invariance model. Metric invariance is established when the model with equality constraints on both item thresholds and factor loadings across groups fits the data comparably to the threshold invariance model. Differences in model fit were evaluated using the scaled χ^2^ difference test, with non-significant results supporting invariance. Moreover, we applied Chen (2007)’s criteria, according to which a difference in CFI ≤ 0.010 and a difference in RMSEA and SRMR ≤ 0.015 indicate substantial equivalence in model fit, even when the χ^2^ difference test is significant. The “lavaan” and “semTools” packages for R were employed to evaluate and compare the different CFA models and to test measurement invariance (Jorgensen et al., 2018; Rosseel, 2012).

Finally, product-moment correlations were calculated to examine the one-month temporal stability of the IUS-P scores, as well as the associations between the IUS-P and the CBCL 6–18 and BRIEF scales (convergent validity). Based on Cohen’s (1998), *r* =.10 indicates a small effect, *r* =.30 a moderate effect, and *r* =.50 a large effect.

## Results

### Factorial Structure 

Table 1 presents the fit indices for the various CFA models of the IUS-P. The one-factor model provided the worst fit. The two-factor model notably improved over the one-factor model, yielding overall acceptable fit indices. However, the high correlation between the Prospective and Inhibitory IU factors (*Φ* = 0.78) raised concerns about the extent to which these two latent variables represent substantially independent constructs (Van Mierlo et al., 2009). The bifactor model further improved the model fit compared to the two-factor model, with excellent fit indices.

**Table 1:** Fit indices for the confirmatory factor analysis models of the IUS-P

	Bifactor model	Two-factor model	One-factor model
χ^2^(*df*)	162.54 (42)*	417.19 (53)*	866.11 (54)*
CFI	0.989	0.966	0.925
TLI	0.982	0.958	0.908
RMSEA	0.060	0.093	0.138
95%CI	0.050-0.070	0.085 − 0.101	0.130 − 0.146
*p*-close	0.042	< 0.001	< 0.001
SRMR	0.030	0.054	0.078

* *p* < 0.001. χ^2^ = scaled chi-square; *df* = degrees of freedom, *CFI* = Comparative Fit Index, *TLI* = Tucker-Lewis Index, *RMSEA* = Root Mean Square Error of Approximation, *95%CI* = 95% confidence interval for Root Mean Square Error of Approximation, *p*-close = RMSEA close fit test, *SRMR* = Standardized Root Mean Square Residual

The standardized factor loadings for the bifactor model are shown in Table 2. All items loaded significantly onto the General factor, with factor loadings ranging from 0.42 to 0.87. With regard to the specific factors, only four out of seven items loaded significantly on the Prospective IU factor (λrange = − 0.10–0.70). In contrast, all Inhibitory IU items loaded significantly on their intended factor (λrange = 0.44–0.62).

**Table 2:** Standardized factor loadings for the bifactor model of the IUS-P

	General	Prospective	Inhibitory
Item #1	0.73**	− 0.05	---
Item #2	0.72**	− 0.10*	---
Item #3	0.45**	0.70**	---
Item #4	0.42**	0.48**	---
Item #5	0.57**	0.11*	---
Item #6	0.87**	− 0.05	---
Item #7	0.82**	− 0.06	---
Item #8	0.67**	---	0.46**
Item #9	0.58**	---	0.62**
Item #10	0.71**	---	0.51**
Item #11	0.64**	---	0.58**
Item #12	0.65**	---	0.44**

* *p* < 0.05; ** *p* < 0.001

The ECV equal to 0.71 met Rodriguez et al. (2016)’s criterion (ECV > 0.70), suggesting a preference for the total score over subscale scores, although the PUC equal to 0.53 recommended caution. Specifically, to determine whether the total score should be favored over subscale scores, model-based reliability coefficients were examined. ω and ωh for the General factor were quite close (0.94 and 0.83, respectively). For Prospective IU, ωh was substantially lower than the corresponding ω (0.042 and 0.88, respectively). Similarly, ωh was remarkably lower than ω for Inhibitory IU (0.36 and 0.92, respectively). These results indicated that the Prospective and Inhibitory IU factors contributed minimally to the reliability of the IUS-P total score, with most of the subscales’ reliability attributable to the General factor variance, particularly for Prospective IU. Overall, these findings suggest that the IUS-P total score is both viable and preferable to the use of separate subscale scores.

### Measurement Invariance

The bifactor model was the baseline model in multigroup confirmatory factor analyses (MGCFA) aimed to test the measurement invariance of the IUS-P across sex (boys: *n* = 408; girls: *n* = 388) and age groups (4–6 years: *n* = 247; 7–8 years: *n* = 285; 9–10 years: *n* = 264). With specific regard to measurement invariance across age groups, a response category (i.e., 5 = *completely agree*) was empty for item #10 in the group of children aged 9 to 10 years; hence, the two extreme response categories (i.e., 4 and 5) for this item were collapsed across all age groups to enable the establishment of thresholds invariance. As shown in Tables 3 and 4, all models demonstrated excellent fit indices in MGCFA analyses by sex and age groups. Additionally, the χ^2^ difference tests between models were non-significant, as well as the differences in other fit indices remained within the recommended thresholds, indicating no substantial loss of fit. Collectively, these findings support the measurement invariance of the IUS-P across both sex and stages of childhood.

**Table 3:** Test of measurement invariance of the IUS-P by sex group

	Configural invariance	Thresholds invariance	Thresholds + Loadings invariance
χ^2^(*df*)	199.75 (84) *	217.53 (108) *	216.36 (129) *
CFI	0.990	0.990	0.992
TLI	0.984	0.988	0.992
RMSEA	0.059	0.051	0.041
95%CI	0.048 − 0.069	0.041 − 0.060	0.031 − 0.051
*p*-close	0.079	0.450	0.934
SRMR	0.034	0.034	0.037
∆χ^2^(*df*)	---	17.78 (24)	−1.17 (21)
∆CFI	---	0	0.002
∆TLI	---	0.004	0.004
∆RMSEA	---	− 0.008	− 0.010
∆SRMR	---	0	0.003

χ^2^ = scaled chi-square, *df* = degrees of freedom, *CFI* = Comparative Fit Index, *TLI* = Tucker-Lewis Index, *RMSEA* = Root Mean Square Error of Approximation, *95%CI* = 95% confidence interval for Root Mean Square Error of Approximation, *p*-close = RMSEA close fit test, *SRMR* = Standardized Root Mean Square Residual, ∆χ^2^(*df*) = chi square difference test, *∆CFI* = change in Comparative Fit Index, *∆RMSEA* = change in Root Mean Square Error of Approximation, *∆SRMR* = change in Standardized Root Mean Square Residual. * *p* < 0.001

**Table 4:** Test of measurement invariance of the IUS-P by age group

	Configural invariance	Thresholds invariance	Thresholds + Loadings invariance
χ^2^(*df*)	244.48 (126) *	283.98 (170) *	290.95 (212) *
CFI	0.986	0.987	0.991
TLI	0.978	0.985	0.991
RMSEA	0.060	0.050	0.038
95%CI	0.048 − 0.071	0.040 − 0.060	0.026 − 0.048
*p*-close	0.078	0.465	0.979
SRMR	0.037	0.037	0.043
∆χ^2^(*df*)	---	39.5 (44)	6.97 (42)
∆CFI	---	0.001	0.004
∆TLI	---	0.007	0.006
∆RMSEA	---	− 0.010	− 0.012
∆SRMR	---	0	0.006

χ^2^ = scaled chi-square, *df* = degrees of freedom, *CFI* = Comparative Fit Index, *TLI* = Tucker-Lewis Index, *RMSEA* = Root Mean Square Error of Approximation; *95%CI* = 95% confidence interval for Root Mean Square Error of Approximation, *p*-close = RMSEA close fit test, *SRMR* = Standardized Root Mean Square Residual, ∆χ^2^(*df*) = chi square difference test, *∆CFI* = change in Comparative Fit Index, *∆RMSEA* = change in Root Mean Square Error of Approximation, *∆SRMR* = change in Standardized Root Mean Square Residual. * *p* < 0.001

### Test-Retest Reliability and Convergent Validity 

First, one-month test-retest reliability of the IUS-P total score was excellent (*r* = 0.67). Subsequently, concerning convergent validity, the correlation matrix between the mean total score of the IUS-P and the mean scores of the other questionnaires is displayed in Table S4 of the Supplementary Information (*see* Online Resource). All correlations were significant and positive. Specifically, the IUS-P demonstrated weak associations with the CBCL 6–18 somatization, attention problems, and aggressive behavior scales, and the BRIEF Inhibit and Plan/Organize scales. Moreover, moderate correlations were observed with the CBCL 6–18 withdrawal/depression and social problems scales, and the BRIEF Emotional control scale. Lastly, strong relations were found with the CBCL 6–18 anxiety/depression scale and the BRIEF Shift scale.

## Discussion

The current research aimed to examine the factorial structure and psychometric properties of the parent-report version of the IUS-R– namely, the IUS-P– in a sample of Italian parents of children aged 4–10 years. The availability of a developmentally appropriate tool enabling early identification of IU is essential, as this transdiagnostic construct has been linked to a broad range of psychological difficulties (e.g., Bottesi, 2023; Iannattone et al., 2023; Osmanağaoğlu et al., 2018). However, assessing IU in this age group presents unique challenges, since children are still developing the cognitive and emotional capacities required to understand and report on complex internal states, particularly abstract constructs such as uncertainty. In this context, a major strength of the IUS-P is that it relies on parental observations, which are particularly important because parents regularly observe their child’s behaviors and emotional responses across various contexts, thus providing a meaningful and ecologically valid perspective. Parent-report tools like the IUS-P are therefore not only practical but also necessary for assessing early manifestations of IU in children who may have limited abilities to self-report their internal experiences in a reliable manner. The present findings support the utility of the Italian IUS-P for this purpose and contribute to the growing body of knowledge on IU in childhood in the following key ways.

### Factorial Structure and Internal Consistency of the IUS-P

The CFAs results showed that the correlated two-factor model had acceptable fit indices. However, the high correlation between the Prospective and Inhibitory IU factors suggested considerable empirical overlap between these dimensions (Van Mierlo et al., 2009). In contrast, the bifactor model offered a significantly better fit to the data, in line with the initial hypotheses and recent research identifying the bifactor model as the best representation of the IUS-R factorial structure in both Italian adults and adolescents (Bottesi et al., 2019, 2023). The bifactor model’s superior fit would indicate that the IUS-P items are influenced by a broad general factor representing the IU trait, along with two narrower group factors that capture the specific dimensions of Prospective and Inhibitory IU.

Pertaining to item loadings on the General factor, they were moderately high and statistically significant. However, two items (i.e., #3 and #4) warrant further attention due to their notably lower – though still moderate – loadings on the General factor. Specifically, these items are distinct in that they require parents to assess their child’s potential thoughts, rather than observable behaviors (i.e., item #3: “He/She might think: ‘People should always think about what might happen. This will ensure that bad things don’t happen.’”; item #4: “He/She might think: ‘Even when you plan things very well, one small detail can ruin everything.’”). Rating these hypothetical, introspective statements may pose challenges for parents, as they require an inference into the child’s internal cognitive processes and thoughts - a task that can be complex, especially for parents with underdeveloped mentalization abilities. In contrast, the other items are more behaviorally oriented, enabling parents to base their responses on direct observation, which is typically easier and more concrete. Hence, the abstract and hypothetical nature of items #3 and #4 may account for their comparatively lower loadings on the General factor. Importantly, it should also be considered that item #3 showed a barely acceptable loading on the General factor in previous studies investigating the IUS-R in both Italian adults (Bottesi et al., 2019) and adolescents (Bottesi et al., 2023). While these studies focused on different age groups and utilized the self-report version of the IUS-R, this recurrent finding suggests that item #3 may not be fully representative of the broad IU construct across different versions of the Italian scale. Thus, the relatively weaker performance of this item may reflect (at least in part) linguistic or cultural nuances that can influence how individuals interpret and respond to the item. Given these concerns, future research should explore the performance of the IUS-P in diverse cultural contexts to determine whether the lower loadings observed for items #3 and #4 are specific to the Italian population or whether they reflect broader issues with these items. Cross-cultural studies would be particularly valuable for clarifying whether linguistic and cultural factors are contributing to the observed discrepancies and whether targeted adaptations to the wording or phrasing of these items could enhance their psychometric performance.

With regard to item loadings on the group factors, the Inhibitory IU factor was rather robust, with all items yielding moderate-to-strong and statistically significant loadings. This suggests that the Italian IUS-P items effectively capture the Inhibitory IU dimension, which reflects the tendency to inhibit or avoid actions and experiences due to uncertainty. In contrast, most IUS-P items did not load significantly on the Prospective IU factor (i.e., items #1, #6, and #7) or exhibited extremely small loadings (i.e., items #2 and #5). This finding mirrors results from several studies employing a bifactor model to examine the factorial structure of the IUS scales in adult, child, and adolescent populations, where the Prospective IU domain consistently appeared weak (e.g., Bottesi et al., 2023; Huntley et al., 2020; Shihata et al., 2018). Generally speaking, these results point to a broader issue with the Prospective IU factor, suggesting that the items designed to measure this specific IU dimension may not fully capture its intended scope in either adults or younger populations. Consequently, further revisions of the items’ content are likely needed to enhance the Prospective IU factor’s clarity and coherence, ensuring that it accurately reflects the cognitive and emotional processes associated with anticipation and preparation for uncertain future events.

Interestingly, item #3 stood out by displaying a high loading on the Prospective IU factor, contrasting with its relatively weaker loading on the General factor. This finding suggests that, in Italian children, item #3 may be the most representative of the Prospective IU dimension. Indeed, this item seems to tap into specific cognitive processes associated with the desire for predictability, such as the need to think and plan ahead to avoid negative, uncertain outcomes. Therefore, it may be particularly suited to capturing the specific nuances of Prospective IU, rather than contributing to the broader IU construct, which encompasses both prospective and inhibitory elements. Further investigation into the specific role of item #3 of the IUS-P, as well as its performance in different populations and cultural contexts, could clarify whether it should be retained as a key indicator of Prospective IU or modified to better align with the broader IU construct.

Subsequently, the present data revealed high ω coefficients for all factors within the bifactor model, reflecting a strong overall reliability of the Italian IUS-P. Notably, while we observed only a slight difference between the ω and ωh coefficients for the General factor, the disparity between these coefficients was much more pronounced for the Prospective and Inhibitory IU factors. This finding suggests that the two group factors contribute minimally to the reliability of their respective subscales. Instead, the majority of the reliable variance is accounted for by the General factor, further underscoring its central role in explaining responses to the IUS-P items. This pattern would indicate that, in the Italian context, the use of a total score is more appropriate than relying on subscale scores for psychological assessment purposes (Rodriguez et al., 2016). Several studies examining the IUS scales in different samples have reached a similar conclusion, favoring the use of a global IU score over separate subscale scores (e.g., Bottesi et al., 2023; Huntley et al., 2020; Shihata et al., 2018). To sum up, although group factors are needed for modeling the IU construct, they hold limited practical relevance for psychological assessment; in contrast, the General IU factor provides a more accurate and meaningful representation of the IU construct, justifying the recommendation to prioritize the total score.

Notably, our study provided robust evidence supporting the measurement invariance of the IUS-P bifactor model across both sex and age groups. Practically speaking, this is an extremely relevant finding, as it ensures that observed differences in the scores on the Italian IUS-P genuinely reflect variations in the IU construct rather than systematic differences in how items are interpreted or responded based on sex or age. As a consequence, the total score of the Italian version of the IUS-P can be reliably used to make inferences across sexes and across the child lifespan. This result lays a strong foundation for future research to expand current knowledge on IU in childhood; in particular, potential directions include exploring sex differences in IU, examining its developmental trajectories, and investigating its role in the onset of psychopathological outcomes. Understanding these aspects will contribute to a more nuanced comprehension of IU’s impact during critical developmental periods and may inform targeted interventions aimed at mitigating its adverse effects.

### Test-Retest Reliability and Convergent Validity of the IUS-P

Our findings indicated good test-retest reliability of the total score of the Italian IUS-P over a one-month period, thus supporting its short-term temporal stability. Consistent with previous evidence on adult populations (e.g., Carleton, 2016), this result suggests that IU may function as a relatively stable trait also during childhood, at least over brief time intervals. Future research employing longer follow-up periods is needed to more thoroughly examine the long-term stability of IU across development. Furthermore, in terms of convergent validity, we observed moderately high positive associations between the IUS-P total score and various internalizing dimensions (i.e., anxiety, depression, social problems, and withdrawal), in keeping with the initial hypotheses. IU has been consistently linked to anxiety and other internalizing disorders in adults and adolescents (e.g., McEvoy et al., 2019; Shapiro et al., 2020); thus, the present results not only confirmed this relation but also extended it to younger populations, highlighting that – in the Italian context – IU is meaningfully associated with a broad range of internalizing features during childhood. Additionally, low but significant positive associations emerged between the IUS-P total score and externalizing features (i.e., aggressive behavior and attention problems). This suggests that IU in Italian children may not be exclusively tied to internalizing dimensions but may also be associated with a wider spectrum of psychopathological features and conditions. By establishing these links, our study contributes to the growing body of literature that highlights IU as a key correlate of both internalizing and externalizing features across the lifespan (Bottesi et al., 2023; Carleton, 2016; Osmanağaoğlu et al., 2018). Although these findings are promising and provide a foundation for future research into the role of IU in the development of externalizing behaviors in children, the weak magnitude of the observed associations precludes definitive conclusions at this stage. However, this should not be interpreted as evidence of limited convergent validity of the Italian IUS-P. Indeed, convergent validity can be supported when associations are consistent in direction and statistically significant, even if effect sizes are moderate - particularly when the variables being compared are related but not redundant (Carlson & Herdman, 2012). Therefore, the weaker associations between IU and externalizing features – especially when compared to the stronger links between IU and internalizing features – likely reflect genuine conceptual distinctions in how IU relates to different domains of child psychopathology, rather than limitations of the Italian IUS-P itself.

### IU and its Relations With Executive Functions

The most innovative findings of the present study pertain to the significant and positive associations emerged between IU and impairments across various executive functioning domains. First, the IUS-P total score was found to be highly related to difficulties in cognitive flexibility. On the one hand, children with high IU levels may struggle with cognitive flexibility due to the pronounced distress provoked by uncertain situations, which may interfere with their ability to adapt and shift between tasks or perspectives. On the other hand, it is also possible that impaired cognitive flexibility contributes to heightened IU levels, predisposing children to experiencing high distress in uncertain situations (Ozsivadjian et al., 2021). For instance, children with low cognitive flexibility tend to engage less in cognitive reappraisal and rely on rigid thinking patterns (Gabrys et al., 2018), which may limit their ability to generate or shift to alternative solutions and interpretation in the face of uncertainty. In particular, this cognitive rigidity may contribute to fostering a perception of uncertainty as threatening or unmanageable, thereby amplifying IU. Essentially, without the cognitive flexibility to reframe uncertain situations in a more adaptive or less threatening way, children may become increasingly intolerant of uncertainty, as they are less equipped to handle the cognitive demands of navigating novel or ambiguous situations.

Subsequently, a moderate association was observed between the IUS-P total score and difficulties in emotional control. Children with high IU levels may struggle to manage their emotional responses, particularly when faced with unpredictable or ambiguous situations; indeed, the negative emotions elicited by uncertainty (i.e., uncertainty distress; Freeston et al., 2020) may overwhelm their capacity for effective emotion regulation, leading to heightened emotional reactivity or challenges in maintaining emotional control (Lauriola et al., 2023; Ouellet et al., 2019; Shu et al., 2022). Simultaneously, however, difficulties in emotional control may further exacerbate IU, as poor emotion regulation can impair children’s ability to process and interpret uncertain situations, thus amplifying the perception of uncertainty as overwhelming or unmanageable (Abbate-Daga et al., 2015).

Finally, the IUS-P total score was found to be weakly associated with impulsivity and difficulties in managing current and future-oriented task demands. As regards impulsivity, children with high IU levels may engage in impulsive behavior as a maladaptive coping strategy to quickly alleviate the negative emotions triggered by uncertainty (Iannattone et al., 2023). Nevertheless, impulsivity may not only serve as a response to IU but could also contribute to a reciprocal dynamic in which efforts to avoid uncertainty through impulsive actions reinforce the perception of uncertainty as undesirable. Over time, this interaction between impulsivity and IU may create a maladaptive cycle, where impulsive behavior and IU co-occur and potentially exacerbate one another (Bottesi et al., 2021). Concerning difficulties in managing current and future-oriented tasks, these may arise from the tendency of children high in IU to focus excessively on negative stimuli and emotions due to worry (Gole et al., 2012). This tendency may divert cognitive resources away from present tasks, disrupting their ability to concentrate on immediate demands. Moreover, persistent worry about uncertain future events may interfere with long-term planning, as children may become overwhelmed by the unpredictability of future outcomes, further hindering their capacity to engage in goal-oriented behaviors (Iannattone et al., 2023). On the other hand, it is also plausible that difficulties in managing current and future-oriented tasks contribute to heightened IU. Indeed, children struggling to effectively organize, plan, or complete tasks may encounter frequent unexpected outcomes, which can reinforce feelings of unpredictability and loss of control. Such difficulties may exacerbate their perception of the future as uncertain and daunting, thus fueling IU.

To sum up, on the basis of the present correlational findings, IU and executive functioning seem reciprocally associated in Italian children. This suggests that interventions focusing on enhancing executive functioning skills in children may help reduce IU-related distress and prevent psychopathological sequelae, as well as interventions addressing IU may enhance executive functioning and support psychological wellbeing. Future longitudinal studies will be crucial in determining whether preventive interventions for children should prioritize targeting IU or executive functioning to achieve optimal outcomes.

### Limitations and Future Directions

Several limitations characterize the present research. First, the sample of parents was mainly composed of mothers, potentially impacting the generalizability of the findings; thus, future studies that strive for a more balanced representation of both parents will be instrumental in capturing the diverse parental perspectives and enhancing the overall validity of the findings. Also, we did not collect data about relevant variables potentially impacting IU, such as race, culture, ethnicity, socioeconomic status, characteristics of the schools, and nature of the living area (e.g., rural or urban). Future research should consider including these dimensions to provide a more comprehensive understanding of how various cultural, environmental, and socioeconomic factors interact with children’s IU. Furthermore, no standard personality trait measures were administered, nor did we include constructs theoretically unrelated to IU (e.g., sensation-seeking). Including these measures in future research could provide a more comprehensive context and improve evidence for divergent validity. Additionally, the study did not include an alternative measure of IU, which limits the ability to directly assess the convergent validity of the IUS-P using a conceptually similar instrument. However, to the best of our knowledge, no developmentally appropriate and psychometrically validated IU measure currently exists for children aged 4–10 years in the Italian context, making such a comparison unfeasible at this time. Therefore, future research would benefit from cross-validation of the IUS-P against newly developed or adapted IU measures for children, as they become available. Importantly, this study relied solely on parent reports of children’s IU without incorporating behavioral assessments. In addition, the use of a single informant across all primary study variables may have contributed to inflated associations due to shared method variance. Future research would benefit from adopting multimodal approaches that integrate behavioral performance and decision-making evaluations in uncertain contexts (Krain et al., 2008), which could complement parent-reported IU data and help identify potential IU subfactors. Including multiple informants (e.g., teacher reports) could also reduce informant bias and further strengthen the construct validity of the IUS-P. Moreover, it is important to note that, while parent-report questionnaires are invaluable with young children, they have limitations. For instance, parental responses may be influenced by the parents’ own emotions, IU levels, stress, or expectations, introducing potential biases into how they perceive and report their child’s thoughts and behaviors. Therefore, administering self-report questionnaires assessing these constructs in parents (e.g., the IUS-R) could provide valuable context and allow researchers to control for these parental factors in their analyses. Similarly, future studies could explore the association between IU and executive functioning by integrating neuropsychological assessments alongside parent reports, thereby providing a more comprehensive understanding of these dynamics. Subsequent studies should also include a sample of parents of children with psychopathology to examine the measurement invariance of the IUS-P across both non-clinical and clinical samples. This would enhance the generalizability of the present results, while also helping pinpoint specific differences in IU relative to the general child population. In this regard, although the present study provided evidence on the link between IU and various psychopathological features, these results should be interpreted within the context of a non-clinical, community-based sample. As such, they offer only initial insight into how IU may relate to early signs of emotional and behavioral difficulties in the general child population. Further research involving clinical samples is thus needed to examine whether and how IU contributes to the onset or maintenance of diagnosable psychological disorders in children. Finally, another important avenue for future research involves establishing empirically derived cut-off scores and interpretative guidelines, which would enhance the direct clinical utility of the IUS-P and support its application in diagnostic and therapeutic settings. Indeed, although the IUS-P holds great promise as a screening tool for assessing IU in children, the current absence of validated cut-off scores limits the precision with which individual results can be interpreted in clinical practice. Therefore, developing normative benchmarks would enable practitioners to better differentiate between typical and potentially problematic levels of IU, thereby improving case formulation, prevention, and intervention planning.

### Conclusions and Practical Implications

Despite the above limitations, our study supported the use of the IUS-P as a reliable tool for assessing IU in Italian children aged 4 to 10 years. This measure provides an opportunity to examine IU in early childhood, facilitating a more comprehensive understanding of IU development across the lifespan. By capturing IU during this critical developmental period, the IUS-P allows researchers and practitioners to investigate its early manifestations and potential long-term impacts, contributing valuable insights to both theoretical research and clinical practice. Indeed, as tentatively demonstrated by the present results, IU seems to have a transdiagnostic role in childhood, being associated with internalizing and externalizing features, as well as with executive dysfunction. This suggests that, under certain individual and environmental conditions, IU may function as a substantial risk factor for the development of emotional, behavioral, and cognitive difficulties early in life. As such, early detection and intervention on IU may play a key role in mitigating its negative impact and improving developmental trajectories.

Practically speaking, although clinical cut-off scores for the IUS-P are not yet available, practitioners may use standardized scores (e.g., *z*-scores) for preliminary screening purposes to identify children who may warrant closer monitoring or more comprehensive assessment. Crucially, high scores should be interpreted with caution, thus not as diagnostic indicators but rather as a potential sign of increased vulnerability due to difficulties in tolerating uncertainty. This information, especially when integrated with other assessment data, may help guide early clinical decisions and implement appropriate interventions.

In this context, the IUS-P may also play a valuable role in enhancing parental involvement, which is often a key component in successful psychological interventions with children (e.g., Pereira et al., 2016). By offering a structured way for parents to reflect on their child’s difficulties with uncertainty, the IUS-P can not only help collect important information but also foster more meaningful parent-child-practitioner collaboration. Beyond screening, the measure may thus serve as a tool for ongoing monitoring and engagement, supporting individualized, parent-inclusive approaches that target IU in early developmental stages.

## Electronic Supplementary Material

Below is the link to the electronic supplementary material.

Supplementary Material 1

## Data Availability

The data that support the findings of this study are available from the corresponding author, Sara Iannattone, upon reasonable request.
