# Supplementary material for: Children Facing the Unknown: An Italian Study Using the Intolerance of Uncertainty Scale– Parent (IUS-P)
Source: Res Child Adolesc Psychopathol. 2025 Aug 5;53(11):1647–61. doi: 10.1007/s10802-025-01355-5 (PMC12586398; doi:10.1007/s10802-025-01355-5)
Supplement: Supplementary file 1 — Supplementary Material 1 [file 10802_2025_1355_MOESM1_ESM.docx]

**Children Facing the Unknown: An Italian Study Using the Intolerance of Uncertainty Scale – Parent (IUS-P)**

**Supplementary Information**

**Table S1.**

*Educational and Employment Status of Mothers and Fathers*

|  | Mothers (%) | Fathers (%) |
| --- | --- | --- |
| Education | | |
| Ph.D. / Specialization diploma / Other certifications | 14.7 | 17.4 |
| Master’s degree | 26.2 | 21.7 |
| Three-year university degree | 15.0 | 8.7 |
| High school diploma | 39.7 | 45.7 |
| Middle school certificate | 4.3 | 6.5 |
| Elementary school certificate | .1 | - |
| Employment | | |
| Employee | 64.6 | 71.7 |
| Self-employed | 10.3 | 14.1 |
| Other roles | 10.0 | 9.8 |
| Homemaker | 9.1 | - |
| Artisian | 2.1 | 3.3 |
| Unemployed | 3.9 | 1.1 |

| **Table S2.**  *Mean raw and T-Scores on the Administered Questionnaires.*   \| Scale \| *N* \| *M* \| *SD* \| Mean T-score \| Range T-score \| \| --- \| --- \| --- \| --- \| --- \| --- \| \| IUS-P \| 796 \| 25.1 \| 8.19 \| - \| - \| \| CBCL - Anxiety/depression \| 596 \| 3.69 \| 3.29 \| 48 \| 38-100 \| \| CBCL - Withdrawal/depression \| 596 \| 1.34 \| 1.73 \| 46 \| 40-93 \| \| CBCL - Somatization \| 596 \| 1.25 \| 1.79 \| 49 \| 42-166 \| \| CBCL - Social problems \| 596 \| 2.31 \| 2.17 \| 50 \| 39-145 \| \| CBCL - Attention problems \| 596 \| 3.11 \| 2.78 \| 45 \| 37-90 \| \| CBCL - Aggressive behavior \| 596 \| 4.00 \| 3.82 \| 43 \| 35-83 \| \| BRIEF - Inhibit \| 629 \| 11.1 \| 2.54 \| 50 \| 36-105 \| \| BRIEF - Shift \| 629 \| 11.0 \| 2.56 \| 49 \| 37-88 \| \| BRIEF - Emotional control \| 629 \| 11.4 \| 3.05 \| 48 \| 36-77 \| \| BRIEF - Plan/Organize \| 629 \| 11.4 \| 2.96 \| 47 \| 35-77 \|   *Note.* IUS-P = Intolerance of Uncertainty Scale – Parent; CBCL = Child Behavior Checklist 6-18; BRIEF = Behavior Rating Inventory of Executive Function.  **Table S3.**  *Mean IUS-P Scores Stratified by age Range and sex.* | | | |  |
| --- | --- | --- | --- | --- | --- | --- | --- | --- | --- | --- | --- | --- | --- | --- | --- | --- | --- | --- | --- | --- | --- | --- | --- | --- | --- | --- | --- | --- | --- | --- | --- | --- | --- | --- | --- | --- | --- | --- | --- | --- | --- | --- | --- | --- | --- | --- | --- | --- | --- | --- | --- | --- | --- | --- | --- | --- | --- | --- | --- | --- | --- | --- | --- | --- | --- | --- | --- | --- | --- | --- | --- | --- | --- | --- | --- | --- |
|  |  | IUS-P | | |
|  |  | *N* | *M* | *SD* |
| Age groups | Preschoolers (4-6 years) | 247 | 24.7 | 7.87 |
|  | Young school-aged (7-8 years) | 285 | 25.3 | 8.45 |
|  | Middle school-aged (9-10 years) | 264 | 25.1 | 8.22 |
| Sex | Boys | 408 | 25.2 | 8.44 |
|  | Girls | 388 | 25.0 | 7.94 |

*Note.* IUS- P = Intolerance of Uncertainty Scale-Parent. No statistically significant differences were found in the IUS-P total score based on age groups (*F* (2, 793) = .382, *p* = .683) or sex (*t* (794) = .323, *p* = .747).

**Table S4**.

*Pearson’s r Correlations Between the IUS-P and the Other Administered Questionnaires.*

|  | IUS-P |
| --- | --- |
| CBCL - Anxiety/depression | .56* |
| CBCL - Withdrawal/depression | .39* |
| CBCL - Somatization | .19* |
| CBCL - Social problems | .38* |
| CBCL - Attention problems | .23* |
| CBCL - Aggressive behavior | .29* |
| BRIEF - Inhibit | .17* |
| BRIEF - Shift | .54* |
| BRIEF - Emotional control | .39* |
| BRIEF - Plan/Organize | .26* |

*Note*. CBCL = Child Behavior Checklist 6-18; BRIEF = Behavior Rating Inventory of Executive Function; IUS-P = Intolerance of Uncertainty Scale – Parent.

* *p* < .001
